# The effects of situated learning and health knowledge involvement on health communications

**DOI:** 10.1186/1742-4755-11-93

**Published:** 2014-12-26

**Authors:** Yi-Chih Lee, Wei-Li Wu

**Affiliations:** Department of International Business, Chien Hsin University of Science and Technology, No.229, Jianxing Road, Zhongli City, Taoyuan County 320 Taiwan

**Keywords:** Framing effects, Situated learning, Health knowledge involvement, Health communication, Visiting a doctor

## Abstract

**Background:**

Many patients use websites, blogs, or online social communities to gain health knowledge, information about disease symptoms, and disease diagnosis opinions. The purpose of this study is to use the online platform of blogs to explore whether the framing effect of information content, situated learning of information content, and health knowledge involvement would affect health communication between doctors and patients and further explore whether this would increase patient willingness to seek treatment.

**Methods:**

This study uses a survey to collect data from patient subjects who have used online doctor blogs or patients who have discussed medical information with doctors on blogs. The number of valid questionnaire samples is 278, and partial least square is used to conduct structural equation model analysis.

**Results:**

Research results show that situated learning and health knowledge involvement have a positive effect on health communication. The negative framing effect and health knowledge involvement would also affect the patient’s intention to seek medical help. In addition, situated learning and health knowledge involvement would affect the intention to seek medical help through communication factors.

**Conclusions:**

Blogs are important communication channels between medical personnel and patients that allow users to consult and ask questions without time limitations and enable them to obtain comprehensive health information.

**Electronic supplementary material:**

The online version of this article (doi:10.1186/1742-4755-11-93) contains supplementary material, which is available to authorized users.

## Introduction

Health communication studies showed that the most effective health communication is customized health communication [1]. The internet is an effective channel for quickly delivering health information. People use websites, blogs, or online social communities to gain health knowledge, information about disease symptoms, and disease diagnosis opinions [2]. Online medical websites alleviate the pressure and fear of visiting a doctor, providing people with sufficient time and a different space to gain the medical information they want at any time. Scholars have noted that most internet users have searched for health information reports online or communicated about health information online [3]. According to the US Harris survey, 93% of people trust online health information, 85% believe that the health information is easy to read, and 82% believe that online health information is of good quality [4]. Therefore, blogs or social networking sites have become new channels for customized health communication.

Framing effects indicate that the presentation of information will affect consumer choices. Information content and framing must conform to the needs, personality traits, reading and writing skills, information literacy, and motivation of individual message recipients [5]. When people make decisions regarding personal purchases, they are often affected by how to describe problems, resulting in different choices [6]. Different information content, descriptions methods, and online arrangements affect consumer behavior [7]. A previous study also has noted that different information framing would affect people’s behaviors toward disease prevention [8]. Therefore, this study puts this construct into the research.

The concept of patient safety is to avoid, prevent, and improve adverse reactions and harm within healthcare processes [9]. Thus, participating in patient safety helps patients obtain necessary medical safety information. Some physicians might lack skills to teach people professional medical terms. They may not have enough time to talk to patients or lack the skills to clearly explain their illness and treatment methods to their patients [10]. That is why there is aloof interaction and poor communication between doctors and patients. As medical treatment processes involve the preserving life, people are concerned with therapeutic effects and self-care. In the education and learning literature, the concept of situated learning has been emphasized in application [11]. Situated learning has become the empirically based educational method and is primarily used with standardized patient training in medical education in Taiwan [12]. Situated learning is a type of acculturation, and the realistic nature of learning activities is an important factor [12]. Previous studies also note that computer technology can be used to assist with instruction, which can help students to understand scientific concepts more easily [13]. Therefore, this study attempts to explore through computer technology how situated learning promotes health education between doctors and patients.

Knowledge is a key factor that affects action, but most people are passive in acquiring health knowledge [14]. People acquire, absorb, and interpret health information based on personal needs or wants, through various media channels [15]. Studies note that the public’s view of diseases not only comes from health information provided by professionals but also from other people’s opinions to organize their own concepts [16]. Scholars have noted that sometimes the public resists or overlooks health information provided by experts. Thus, experts should listen to the voices of the people and begin a bilateral dialogue, to achieve effective transmission of health information [17]. In addition, individual behavioral involvement would affect attitude, which can be used to predict intention [18]. When people increase health care knowledge, they have greater concerns about their health, and they understand the importance of prevention, which may also elevate their intention to seek medical treatment.

The purpose of this study is to use the online platform of blogs to explore whether the framing effects, situated learning of information content, and health knowledge involvement would affect health communication and to further study whether this exploration would elevate the patient’s intention to seek medical treatment. This study attempts to provide theoretical and empirical results for improving the relationship between doctors and patients. We propose the following hypotheses:

Hypothesis 1: Positive or negative information content affects health communication.

Hypothesis 2: Positive or negative information content affects one’s intention to seek medical treatment.

Hypothesis 3: Online situated learning is positively related to health communication.

Hypothesis 4: Online situated learning is positively related to one’s intention to seek medical treatment.

Hypothesis 5: Health knowledge involvement is positively related to health communication. The greater the extent that patients are involved with health knowledge, the more effective the health communication they receive will feel.

Hypothesis 6: Health knowledge involvement is positively related to one’s intention to seek medical treatment. The greater the extent that patients are involved with health knowledge, the more inclined they will be to seek medical treatment.

Hypothesis 7: Health communication is positively related to one’s intention to seek medical treatment.

Hypothesis 8: Health communication mediates the effect of framing effect on one’s intention to seek medical treatment.

Hypothesis 9: Health communication mediates the effect of situated learning on one’s intention to seek medical treatment.

Hypothesis 10: Health communication mediates the effect of health knowledge involvement on one’s intention to seek medical treatment.

## Methods

### Sample and ethical clearance

The data for this study were collected from Aug 1, 2012 to Jun 30, 2013. This study established the stimulated blog experiment. We recruited survey participants who were seeking medical treatment in a clinic or hospital in Taiwan. Participants were asked to indicate their agreement with statements. There were 278 valid questionnaire samples in this study. The participants had to be adults and were informed that their participation was voluntary. This study did not collect the participants’ privacy, and the informed consent was obtained from all of the participants. Ethical considerations in this study had reported to an appropriate authority. Their average age was 33.6 years, of which 63.1% females and 69.3% college graduates.

### Measures

The experiment design variable in this study is the framing effect, divided into positive and negative goal framing. The study refers to the Rothman and Salovey (1997) method to revise the weight loss issue to form the goal framing of this study [19]. The content of a positive goal framing enhances the benefits brought by weight loss; the content of a negative goal framing enhances the loss caused by failure to participate in weight loss. In the situated learning variable, blogs provide situated learning texts for social interaction. This study built on the findings by Brown et al. (1989) to develop a scale suited to the medical scenario, with seven points measured using a seven-point Likert scale (1 = highly disagree, 7 = highly agree) for testing [20]. Health involvement primarily referred to questionnaires by Lee (2013) [21]. There were a total of three questions, “…to me, this health information is influential”, “…related”, and “…meaningful”.. Regarding the construct of health communication, this study defines it as a patient’s communication in medical consultations to ask more questions, express opinions, and understand medical information; the scale is modified from Hyun Jung Oh & Byoungkwan Lee (2012), with a total of four questions [22]: “Health issues discussed about on this medical blog are easy to understand”, “…I clearly know how to raise a question effectively”, “I can understand the type of disease from the doctor’s responses”, and “…I can acquire medical information I need from blogs”. Finally, regarding intention to seek medical treatment; Lin et al. (2009) uses three questions, “…The doctor’s recommended weight loss method is worth considering;” “…I am willing to participate in treatment;” “…I’d like to go to a doctor’s outpatient clinic for consultation” , to determine whether after people use blogs to consult with doctors or seek medical information, they are willing to visit doctors [23]. This study uses Partial Least Square (PLS) to conduct reliability and validity testing, as well as overall model analysis. The software used is smartpls, with overall reliability and validity as shown in Table 1. The constructs of the measurement model have good reliability, with composite reliability greater than 0.7, and average variance extracted (AVE) over 0.5, both of which reach the standard for convergent validity.

**Table 1:** **The fit index of the model**

Construct	Composite reliability	AVE	Cronbach's alpha	Situated learning	Health involvement	Health communication	Intention to seek medical treatment
Health communication	0.90	0.57	0.88	0.76			
Intention to seek medical treatment	0.93	0.81	0.88	0.41	0.90		
Situated learning	0.79	0.56	0.70	0.45	0.31	0.75	
Health Involvement	0.92	0.76	0.83	0.35	0.33	0.50	0.87

## Results

This study established 10 hypotheses. The hypotheses were confirmed using PLS for structural equation analysis, with the structural model results as shown in Table 2. Standardized coefficient and significance testing show that patients who accept negative framing have more effective medical communication (Estimate value = -0.038, p = 0.292), but this is not statistically significant, as H1 is not supported; however, when patients have negative descriptions of blog information, their intention to seek medical treatment (Estimate value = -0.1578, p = 0.001) is elevated, thus, H2 is supported. Research results show that when patients accept negative goal framing, their intention to seek medical treatment is affected, but their intention not to seek communication is affected. In the situated learning construct, when patients use blog descriptions of interaction between doctors and patients or among patients and gain relevant information, they are more affected by communication (Estimate value = 0.3652, p < 0.001). H3 is supported; however, this does not elevate patient intention to seek medical treatment (Estimate value = 0.0472, p = 0.374), and H4 is not supported. Another result of this study shows that although situated learning can improve communication, the effect on intention to seek medical treatment is not statistically significant. In the construct of health involvement, when patients more frequently absorb health knowledge, they are more affected by communication (Estimate value = 0.1693, p = 0.005). Simultaneously, their intention to seek medical treatment is greater (Estimate value = 0.2105, p = 0.001); thus, H5 and H6 are supported. The third construct shows that when patients are highly involved with health knowledge, both their health communication and intention to seek medical treatment are elevated. When people communicate more on the blog, they are more willing to see a doctor (Estimate value = 0.3046, p < 0.001). The model in this study has an average of R^2^ = 0.43, which is significantly greater than 0.35 [24], GOF = 0.55; thus, the model has good fit.

**Table 2:** **The results of the structural equation model**

Structural path		Estimate value	t value	p value	Verify
H1	Framing effect → Health communication	-0.038	1.0564	p = 0.292	No Support
H2	Framing effect → Intention to seek medical treatment	-0.1578	3.2161	p = 0.001	Support
H3	Situated learning → Health communication	0.3652	6.3941	p < 0.001	Support
H4	Situated learning → Intention to seek medical treatment	0.0472	0.8911	p = 0.374	No Support
H5	Health Involvement → Health communication	0.1693	2.8019	p = 0.005	Support
H6	Health Involvement → Intention to seek medical treatment	0.2105	3.3370	p = 0.001	Support
H7	Health communication → Intention to seek medical treatment	0.3046	5.068	P < 0.001	Support

p < 0.05 significant.

To further examine the mediating hypotheses, this study uses Sobel testing to confirm the mediating effect [25]. According to Preacher and Hayes (2004) [26], the method is computed based on the path coefficient and standard error. When the Z value is greater than 1.96, it means there is a significant mediating effect, showing that health communication does not play a mediating role between framing effect and intention to seek medical treatment; thus, H8 is not supported. However, health communication plays a mediating role between situated learning and intention to seek medical treatment (z = 3.9722, p < 0.001) and between health involvement and intention to seek medical treatment (z = 2.4528, p = 0.014), with results shown in Table 3.

**Table 3:** **Sobel test’s table**

	Mediating path	Sobel z test	p value
H8	Framing effect → Health communication → Intention to seek medical treatment	-1.0361	0.300
H9	Situated learning → Health communication → Intention to seek medical treatment	3.9722	<0.001
H10	Health Involvement → Health communication → Intention to seek medical treatment	2.4528	0.014

p < 0.05 significant.

## Discussion

This was a pilot study to test the effectiveness of message framing, situated learning, and involvement of public health knowledge on a blog platform for health communication. Furthermore, it investigated the influences of those factors on people’s willingness to seek medical attention. The research results showed that, firstly, when a blog used a loss-framed message to describe medical information, people’s willingness to seek medical attention is increased. Secondly, a blog’s provision of situated learning would have a positive effect on people’s information communication, and increase their willingness to seek medical attention. Thirdly, people would have a better understanding of medical information if they have a higher level of health knowledge, and their willingness to seek medical attention would increase accordingly.

This study attempted to investigate whether people’s willingness to receive medical care would be affected by gain and loss frames. The empirical results showed that the use of loss-framed messages on medical information in the blog had an effect on people’s willingness to seek medical attention. In the goal framing, this study focused on the relationship between engaging or not engaging in a certain behavior and achieving/not achieving an objective. According to the data from the WHO, obesity leads to higher mortality. In addition, 44% of obese people suffer from diabetes, 23% suffer from cardiovascular diseases, and 7-41% suffer from cancer [27]. Therefore, people regard not engaging in weight loss behavior as a negative loss, more specifically, representing the failure to maintain health or even affect the life. Therefore, this result showed that loss-framed messages had a stronger driving force affecting people’s willingness to seek medical attention. This finding was consistent with Pakpour’s study [28].

Another focus of this study was on the inclusion of situated learning. This study included simulated training models of patients/cases, as well as various possible medical care situations in clinical practice to enable the patients to learn self-care abilities and skills in realistic simulations of interactive situations. The empirical results showed that situated learning was beneficial to health communication and could improve patients’ health care ability. This result was consistent with that of Aadal (2011) [29]. In past studies, situated learning was applied to the educational training of clinical doctors, nursing personnel and medical students to simulate patients’ problems and present them as cases, as well as accurately convey medical care knowledge [30–34]. However, as the concept of patient safety encourages patients to participate in their own healthcare, it is important for doctors to use situated learning to deliver difficult medical knowledge regarding the processes of medical actions and techniques in later healthcare. There are discussions regarding disease treatment and topics regarding emotional management on online forums; however, people cannot know whether such advice is correct. Medical personnel have professional clinical knowledge and can use previous case experience to accumulate information on issues that tend to occur for patients and clearly describe the clinical care processes of these cases, which enhances the confidence of patients in the medical process and dispels needless fear, which can promote good communication between doctors and patients. Other than medical personnel, it is also possible to provide senior patients with quality counseling, thus, enhancing patient confidence through empathy. Through situated learning, patients are able to conduct basic self-care and alleviate their doubts regarding doctor treatment, which encourages them to continue to seek medical treatment.

Goodman (1995) noted that health involvement can change the knowledge of the public [35]. This study shows that when patients have high health knowledge involvement, their health communication effects are better. This result is similar to Nielsen’s study (2014) which pointed out that there was significant correlation between level of knowledge and health-seeking behavior [36]. This study also revealed that when people more actively seek involvement with health knowledge and are adept at searching online for medical information, they have greater understanding of online medical information. Simultaneously, because these people place greater emphasis on their own health, when there are health problems, they have a greater to intention to seek medical treatment.

The main contribution of this study was to apply the concept of situated learning, which tends to be used to teach clinical medical and nursing personnel and students using stimulation training and skills, to the health education of patients. Situated learning is different from traditional learning approaches. It provides convenient learning, thus improving the effectiveness of health communication and further increase patients’ willingness to seek medical attention. This study verified these outcomes. The findings of this study have important implications for clinical medical and nursing personnel to run network platforms. Firstly, when people face medical problems, they simplify messages to interpret medical outcomes [37]. Therefore, doctors on blogs have to encourage people to seek medical attention. Doctors should also use people’s fear of life-threatening risks to strengthen the information on the risks of illness. In this way, future medical disputes can be avoided, and people can be encouraged to seek medical attention as early as possible. Next, patients need to be educated. With limited clinical hours and placements, doctors cannot fully convey all of the medical information. With the application of computer technology, complicated medical knowledge can be converted into various medical cases to educate people through various situations. Chang and Liu (2001) suggested that multimedia learning materials incorporating sound, pictures, animation and video, could encourage learners to keep a long-standing interest in watching and learning [38]. Situated learning can use interactive multimedia applications to enable patients easily to learn through the internet at anytime and anywhere to make health education more interesting. In addition to reducing the medical risks faced by people, it can also improve their understanding of professional medical terms, which is greatly contributive to their self-health care and health literacy. Moreover, the waiting time at many clinics is quite long and boring for patients. Medical and nursing personnel should use the popularity of wireless internet technology and smart phones to provide health communication early before the patients’ appointments, which will be beneficial to patients’ understanding of the clinical enquiry process.

## Research limitations and future studies

The explanatory model of this study can be expanded upon. In addition, the measurement items in the constructs may incorporate more constructs for comparison with the medical information transmission of blogs set by medical personnel, as well as other websites and social media websites. Because this study was constrained by research issues and design, it is suggested that future research engage the following topics. First, the information text and situated learning content in this study both focus on the issue of weight loss. Because there are various types of medical departments, patients have a wide range of concerns; thus, it is suggested that future researchers incorporate diseases of different severity to measure whether information presentation has an effect on how patients understand information. Second, individual explanatory styles can possibly affect communication effects and intention to seek medical help. For instance, if people are pessimistic and have a negative understanding of disease treatment, it would obstruct medical information communication and transmission and may even cause them to reject medical help. These are topics worthy of future exploration by researchers.

## Conclusions

The convenience of the internet is an important communication channel that reduces the information gap between medical personnel and patients. As disseminators of health information, medical personnel can use the online platform to send various types of health information to people who need it [39]. Most importantly, patients can consult with online medical personnel autonomously and ask questions without fear, obtaining all of the information on health that patients want to know.

## Authors’ original submitted files for images

Below are the links to the authors’ original submitted files for images. Authors’ original file for figure 1
