# Supplementary figures and images for: The effects of situated learning and health knowledge involvement on health communications
Source: Reprod Health. 2014 Dec 26;11:93. doi: 10.1186/1742-4755-11-93 (PMC4297390; doi:10.1186/1742-4755-11-93)

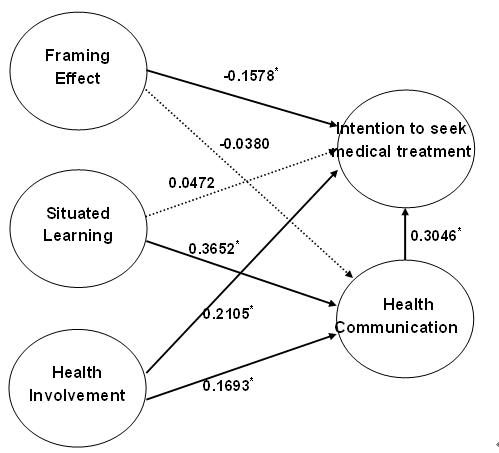

Supplement: Supplementary file 1 — Authors’ original file for figure 1 [file 12978_2014_341_MOESM1_ESM.tiff]
